# Rapid quantification of 50 fatty acids in small amounts of biological samples for population molecular phenotyping

**DOI:** 10.52601/bpr.2023.230042

**Published:** 2023-12-31

**Authors:** Pinghui Liu, Qinsheng Chen, Lianglong Zhang, Chengcheng Ren, Biru Shi, Jingxian Zhang, Shuaiyao Wang, Ziliang Chen, Qi Wang, Hui Xie, Qingxia Huang, Huiru Tang

**Affiliations:** 1 State Key Laboratory of Genetic Engineering, School of Life Sciences, Human Phenome Institute, Zhangjiang Fudan International Innovation Center, Metabonomics and Systems Biology Laboratory at Shanghai International Centre for Molecular Phenomics, Zhongshan Hospital, Fudan University, Shanghai 200032, China; 2 Wuhan Laboratory for Shanghai Metabolome Institute (SMI) Ltd, Wuhan 430000, China

**Keywords:** Fatty-acidomics, High-throughput quantification, Structure-retention relationship, No-additive retention index

## Abstract

Efficient quantification of fatty-acid (FA) composition (fatty-acidome) in biological samples is crucial for understanding physiology and pathophysiology in large population cohorts. Here, we report a rapid GC-FID/MS method for simultaneous quantification of all FAs in numerous biological matrices. Within eight minutes, this method enabled simultaneous quantification of 50 FAs as fatty-acid methyl esters (FAMEs) in femtomole levels following the efficient transformation of FAs in all lipids including FFAs, cholesterol-esters, glycerides, phospholipids and sphingolipids. The method showed satisfactory inter-day and intra-day precision, stability and linearity (R^2^ > 0.994) within a concentration range of 2–3 orders of magnitude. FAs were then quantified in typical multiple biological matrices including human biofluids (urine, plasma) and cells, animal intestinal content and tissue samples. We also established a quantitative structure-retention relationship (QSRR) for analytes to accurately predict their retention time and aid their reliable identification. We further developed a novel no-additive retention index (NARI) with endogenous FAMEs reducing inter-batch variations to 15 seconds; such NARI performed better than the alkanes-based classical RI, making meta-analysis possible for data obtained from different batches and platforms. Collectively, this provides an inexpensive high-throughput analytical system for quantitative phenotyping of all FAs in 8-minutes multiple biological matrices in large cohort studies of pathophysiological effects.

## INTRODUCTION

Fatty acids (FAs) are important nutrients for all organisms with numerous physiological functions (Bazinet and Layé 2014; Dalile *et al.* 2019; Röhrig and Schulze 2016) and quantitative analysis of FA composition (fatty-acidome) in small amounts of biological samples in large population cohorts is essential for understanding such functions (Losito *et al.* 2018; Wang *et al.* 2013). FAs can generally be categorized into saturated fatty-acids (SFAs) and unsaturated ones (UFAs) including monounsaturated fatty-acids (MUFAs) and polyunsaturated ones (PUFAs); the latter are essential FAs for mammals including both n3 and n6 ones (Wall *et al.* 2010). In biological samples, these FAs are present in the forms of free fatty-acids (FFAs) and esterified FAs such as phospholipids, sphingolipids (SPs), glycerides, cholesterol-esters (CEs), FA-esters of hydroxyl-FAs (FAHFAs) and acylcarnitines (ACars) (Fahy *et al.* 2005; Han 2016; Zhang *et al.* 2023). In human blood plasma and serum, for example, FAs are present as FFAs and lipoproteins implicated in numerous pathophysiological processes (Chen *et al.* 2020, 2023; Li *et al.* 2015b, 2023a; Loo *et al.* 2021; Nicholson 2021; Wu *et al.* 2021b; Xia *et al.* 2021, 2022). The biological functions of FAs and their metabolites are diverse including cellular membrane homeostasis (Romero *et al.* 2019), energy generation (Lopaschuk *et al.* 2010), regulation of transcription-factor activity (Zhang *et al.* 2021) and signaling (Dalile *et al.* 2019). For instance, short-chain FAs play vital roles in not only anti-inflammation but also regulation of glucose and lipid homeostasis (Frampton *et al.* 2020; Hummasti and Hotamisligil 2010; Wang *et al.* 2018; Wu *et al.* 2021a) whilst FA acylation of proteins had vital pathophysiological implications (Dai *et al.* 2020; Zhu *et al.* 2021). FAs in various forms were further considered as indicators for nutritional status, cardiometabolic and neurodegenerative diseases as well as cancer (Kim 2018; Li *et al.* 2023a; Lin *et al.* 2010; Nicholson 2021; Wu *et al.* 2021b; Xia *et al.* 2021, 2022). For example, a biomarker set consisting of eight fatty acids effectively indicated cell viability and characterized the hepatotoxicity of amiodarone (Li *et al.* 2023b). Biological functions of FAs are continuously emerging but far from fully understood whilst only small amounts of biological samples are often available. Therefore, high-throughput methods for quantifying fatty-acid composition (fatty-acidomics) in small amounts of samples are still essentially required especially for large cohort studies.

## REVIEW

Quantification of FAs is classically done using GC-FID/MS (Agnew *et al.* 2019; An *et al.* 2013; Ecker *et al.* 2012; Han *et al.* 2011; Huang *et al.* 2019b; Ichihara and Fukubayashi 2010; Jiang *et al.* 2017; Masood *et al.* 2005; Tremblay-Franco *et al.* 2015; Xia *et al.* 2019; Xu *et al.* 2010; Zhang *et al.* 2015; Zhao *et al.* 2017), LC-MS (Han *et al.* 2015; Jiang *et al.* 2017; Mattarozzi *et al.* 2021; Wang *et al.* 2013) and NMR (Cai *et al.* 2017) methods. GC-FID/MS is clearly the technology of choice with its high sensitivity, capability of separating positional and cis-trans isomers, excellent stability and availability of databases for fatty-acid-methyl-esters (FAMEs). This is achieved by measuring FAMEs resulting from transesterification (Ostermann *et al.* 2014) catalyzed with bases or acids. The former include potassium hydroxide (KOH-MeOH) (Huang *et al.* 2019b) and sodium methoxide (NaOCH_3_) (Glaser *et al.* 2010) whereas the latter include boron trifluoride (BF_3_-MeOH) (Bondia-Pons *et al.* 2007), methanolic hydrochloric acid (HCl-MeOH) (Ichihara and Fukubayashi 2010), methanolic sulfuric acid (H_2_SO_4_-MeOH) (Agnew *et al.* 2019) and acetyl-chloride (CH_3_COCl-MeOH) (An *et al.* 2013; Li *et al.* 2015a; Xu *et al.* 2010; Zhang *et al.* 2015). However, bases are ineffective for methylation of FFAs and SMs whilst BF_3_-MeOH is unstable and toxic (Bondia-Pons *et al.* 2007); HCl-MeOH and H_2_SO_4_-MeOH are inconvenient to prepare and by-products can be formed at high-temperature (Agnew *et al.* 2019).

CH_3_COCl-MeOH appeared to be the choice of method. However, the existing methods often required a dozen hours for methylation and dozens of minutes for each spectral acquisition (An *et al.* 2013; Li *et al.* 2015a; Xu *et al.* 2010; Zhang *et al.* 2015) limiting their applications in large cohort studies. Even the fastest GC-FID/MS method required 15-min for data acquisition but detected only 24 endogenous FAs in human plasma samples (Ecker *et al.* 2012) without covering some important FAs (*e*.*g*., C11:0, C14:1n5c, C16:1n9c, C17:1n7c, C20:2n6c, C22:1n9c, C22:3n3 and C23:0). For instance, the levels of C14:1n5c and C16:1n9c in plasma were reported as important diagnostic indicators for long-chain fatty-acid oxidation defects (Cecatto *et al.* 2020) and Meniere's disease (Coon *et al.* 2023); C22:1n9 is a possible therapeutic agent for neurodegenerative diseases (Goyal *et al.* 2023). Some other methods with derivatization at high temperatures (95–100 °C) might impair analytical sensitivity and accuracy due to isomerization and instability of UFAs (Chiu and Kuo 2020; Liu *et al.* 2018). These methods also demand sizable samples which are not always available and transesterification efficiency for all fatty-acid forms needs clarifying together with the temperature-dependent isomerization for UFAs. Furthermore, additives (alkanes) were classically used to generate a retention index (RI) (Kovats 1958) to correct the batch effects universally present in chromatographic separation. Unfortunately, such additive-based RI methods inevitably introduce extra unwanted matrix effects and ionization interferences, especially for analytes coeluted with the additives. Moreover, quantitative structure-retention relationships (QSRR) remain to be established for predicting the retention time (*t*_*R*_) of analytes when their standards are unavailable.

To address these, here, we developed a parameter-optimized transesterification method using CH_3_COCl-MeOH and ensured complete methylation of FAs in all important forms of lipids. We then developed a rapid GC-FID/MS method for simultaneous quantification of all FAs in multiple biological matrices with an 8-min data acquisition. We further established a no-additive retention index (NARI) for FAMEs to correct the batch effects and a QSRR model for predicting *t*_*R*_ of FAMEs with standards unavailable.

## RESULTS AND DISCUSSION

Large cohort studies of lipid metabolism require high-throughput quantification of fatty acid composition in different biological samples with minimum inter-batch variations. To meet this demand, we established an 8-min GC-FID/MS method for simultaneously quantifying 50 FAs in multiple biological matrices with coverage of all lipid types. This has much higher coverage and is more rapid than previous methods (An *et al.* 2013; Li *et al.* 2015a; Xu *et al.* 2010; Zhang *et al.* 2015). We also developed a novel no-additive retention index (NARI) for correcting inter-batch variations with better performance than the alkanes-based classical RI (Kovats 1958). We have further established a quantitative structure-retention relationship (QSRR) for predicting *t*_*R*_ of FAMEs when their standards are unavailable.

### Efficient transesterification of fatty acids in different lipids and GC-FID/MS quantification

**Figure 1:**
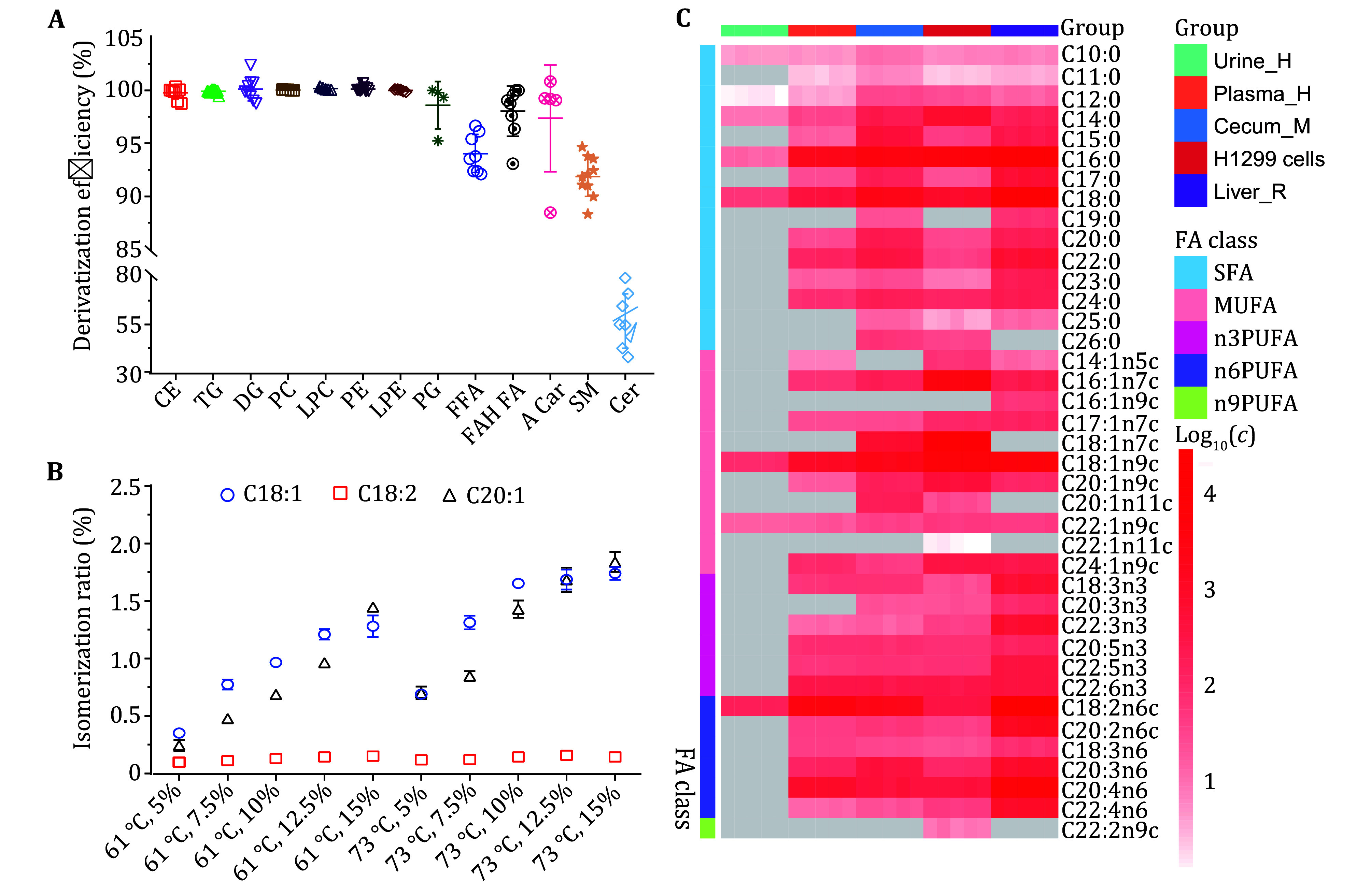
High-throughput quantitative fatty-acidomics analysis. **A** Transmethylation efficiency (12.5% CH_3_COCl, 73 °C, 3 h) for different lipids including cholesteryl-esters (CE), triglycerides (TG), diglycerides (DG), lysophosphatidylcholines (LPC), phosphatidylcholines (PC), lysophosphatidylethanolamines (LPE), phosphatidylethanolamines (PE), phosphatidylglycerol (PG), free fatty acids (FFA), fatty acid esters of hydroxyl-fatty-acids (FAHFA), acylcarnitines (ACar), sphingomyelins (SM) and ceramides (Cer). **B** Isomerization of some representative unsaturated fatty acids as a function of temperature and CH_3_COCl concentration. C concentrations (*c,* μmol/L) of fatty acids in 10 μL human fluids (urine and plasma) and 10 mg of other biological matrices (H1299 cells, mouse cecum content and rabbit liver tissue)

The efficient transformation of FAs in all lipids into FAMEs is the prerequisite for their high coverage quantification using GC-FID/MS. MeOH-CH_3_COCl was proven as a mild and safe transesterification reagent (An *et al.* 2013; Li *et al.* 2015a; Xu *et al.* 2010). However, the transesterification efficiency for fatty acids in different types of lipids remains to be clarified. Therefore, we here systematically optimized parameters including CH_3_COCl concentration, reaction time and temperature using human blood plasma samples. Differing from what was reported previously (Ecker *et al.* 2012; Lepage and Roy 1986), our optimal reaction parameters were 12.5% CH_3_COCl, 73 °C, 3 h and 6:1 for MeOH/hexane ratio (*V/V*) enabling methylation of the above 95% lipids (Fig. 1A). Only <2% isomerization by-products were detected for some UFAs with different carbon chain-lengths having one and two double bonds (Fig. 1B). Notably, our results also showed that the levels of isomerization by-product from C18:2n6c was obviously lower than those from C18:1n9c (Fig. 1B) being consistent with previously reported results that PUFAs with *cis-*fatty acids were less prone to isomerization than monosaturated FAs under same derivatization conditions (Agnew *et al.* 2019). This avoided the temperature-induced isomerization for UFAs (Liu *et al.* 2018) and acid-hydrolysis of FAMEs (Chu *et al.* 2015) above 80 °C thus clearly had better methylation performance than the reported methods (Ichihara and Fukubayashi 2010; Xu *et al.* 2010) (supplementary Fig. S1–S3). Such was confirmed here with an LC-MS method (Huang *et al.* 2019a; Loo *et al.* 2021) by monitoring 13 lipid subclasses including TG, PC, LPC, PE, LPE, PG, FFAs, sphingolipids (SM), CE, DG, ceramides (Cer), FAHFA and acyl-carnitines (Fig. 1A, supplementary Figs. S2 and S3) unlike previous studies monitoring only TG, phospholipids, sphingolipids, FFAs and CE (Ichihara and Fukubayashi 2010; Xu *et al.* 2010). Notably, ceramides showed about 55% derivatization probably due to the resistance of their amide-bonds to acid-catalyzed transmethylation (Masood *et al.* 2005). However, ceramides only account for less than 1% of fatty acids in biological samples.

**Figure 2:**
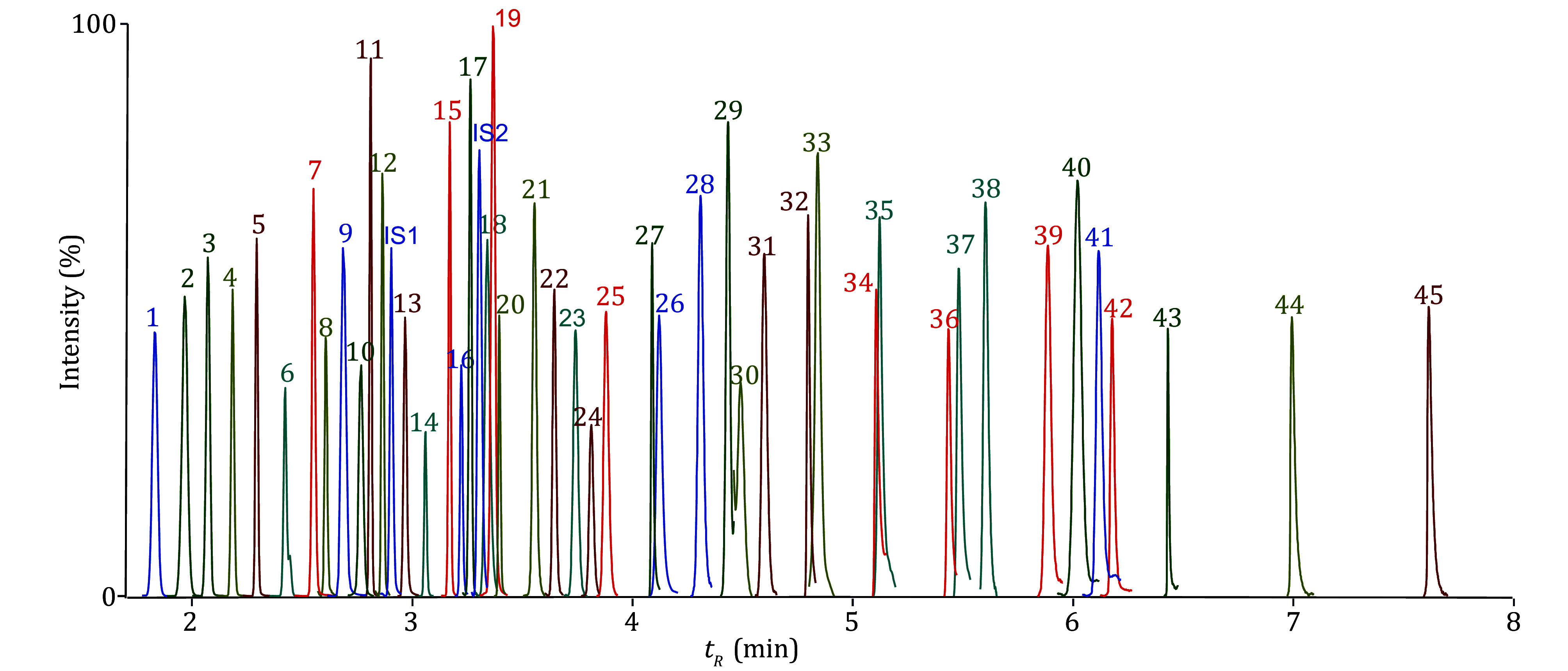
Total ion chromatogram (TIC) from GC-FID/MS analysis of 45 methylated FAs (Keys for analytes are listed in supplementary Table S3)

Furthermore, simultaneous quantification of FAs was achieved here with an 8-min acquisition with our comprehensively optimized GC-FID/MS parameters (supplementary Tables S1 and S2) showing good separation of UFA isomers such as C18:3n6-C18:3n3 and C18:2n6t-C18:2n6c (Fig. 2). This enables measurements of FA ratios as indicators of enzymes such as SCD1 (C16:1/C16:0) and elongases (C18:0/C16:0). This method also has higher throughput and coverage than all previous ones (An *et al.* 2013; Ecker *et al.* 2012; Gao *et al.* 2009; Garlito *et al.* 2019; Han *et al.* 2011, 2015; Jiang *et al.* 2017; Lepage and Roy 1986; Li *et al.* 2015a; Masood *et al.* 2005; Tremblay-Franco *et al.* 2015; Xia *et al.* 2019; Xu *et al.* 2010; Zhao *et al.* 2017) (supplementary Table S3). Since data acquisition is the speed-limiting step, this rapid analysis provided a feasible approach for large cohort studies when combined with automatic sample-preparation robotics. Moreover, this method had excellent linearity (R^2^ > 0.994) for all analytes in a concentration range of 2–3 orders of magnitude with LOD (S/N~3) and LOQ (S/N~10) below 20 and 40 fmol (as low as 0.5 and 2 fmol) on the column, respectively (supplementary Table S4). For all three concentration levels, the intra- and inter-day CV values were below 20% (below 15% for 95% analytes) with recoveries of 80%–120% (supplementary Table S5) and good stability (CV < 15%) in autosampler (20 °C) and storage at 4 °C and –80 °C (supplementary Table S6) except few compounds whose concentrations were close to LOQ.

### Applicability for quantifying fatty-acids in multiple typical biological matrices

We confirmed the applicability of this method for quantifying fatty acids in multiple typical biological matrices including human urine, plasma, cells, animal intestinal content and liver tissue samples (supplementary Table S7). The results showed significant differences in FA composition hence molecular phenotypes for these biological samples (Fig. 1C). The composition of FAs in human plasma was broadly consistent with what was reported (Masood *et al.* 2005). However, our method managed to quantify 30 FAs in healthy human plasma (supplementary Table S7) including some low-level ones (C11:0, C17:1n7c, C22:3n3 and C23:0) compared to less than 22 FAs with other methods (Han *et al.* 2011; Tremblay-Franco *et al.* 2015).

Some interesting molecular phenotypic features were observable for fatty acids in these biological samples. Whilst mouse cecum contents contained rich SFAs, human non-small cell lung cancer cells H1299 had more MUFAs whereas rabbit liver samples contained rich SFAs and PUFAs. Much fewer fatty acids were detectable in urine with only a few saturated (C10:0, C12:0, C14:0, C16:0, C18:0) and unsaturated fatty acids (C18:1n9c, C18:2n6c, C22:1n9c). In contrast, cecum content samples contained noticeably more odd-carbon SFAs (C15:0, C17:0, C19:0, C23:0, C25:0) than plasma and mammalian cell samples (supplementary Table S7). This is understandable and probably due to gut microbial contributions. However, these odd-carbon SFAs also had higher levels in rabbit liver tissue than human plasma and cells implying some profound effects of dietary and gut microbial FAs on the mammal physiology since conventional wisdom believed that mammals were only capable of synthesizing the even-carbon FAs. If this conventional belief were correct, then the odd-carbon fatty acids in human plasma detected here (C11:0, C15:0, C17:0, C23:0) should also be from dietary and gut microbiota whereas these in human cells (C15:0, C17:0, C23:0, C25:0) should be from their culture media, requiring further investigation. Such arguments about the origins and functions of odd-carbon fatty acids are clearly beyond the scope of this study but warrant further investigations. Nevertheless, these verified the applicability of the current method to multiple biological matrices for new discoveries in terms of nutritional and physiological effects. Since not all fatty acids have commercial standards, it is essential to establish empirical models for quantitative structure-retention relationships to assist reliable identification of analytes without standards and for correcting inter-batch variations in order to compare data from different batches, especially for large cohort studies.

### Quantitative structure-retention relationship and no-additive retention index for FAMEs

**Figure 3:**
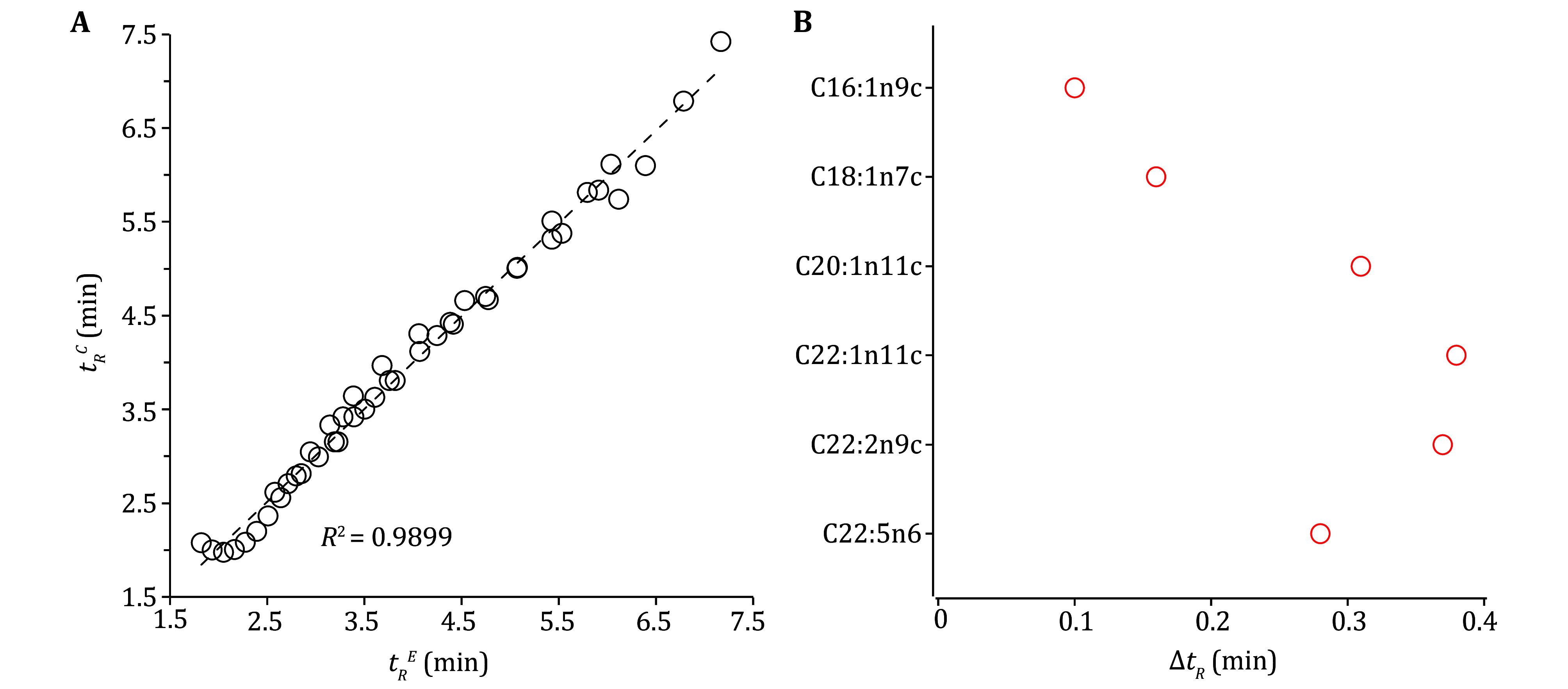
Correlations for experimental (*t*_*R*_^*E*^) and calculated values (*t*_*R*_^*C*^) from the quantitative structure-retention relationship (**A**) and prediction accuracy for six test analytes (**B**)

Quantitative structure-retention relationship (QSRR) for analytes is vital for predicting their retention time hence assisting their reliable identification for chromatography-based analysis (Héberger 2007; Hellmuth *et al.* 2012; Hu *et al.* 2022; Kaliszan 2007; Ovčačíková *et al.* 2016; Vu *et al.* 2019); numerous calculated structural descriptors were considered in literature (Héberger 2007; Kaliszan 2007) though such methods were less practical. Interestingly, our experimental *t*_*R*_ results clearly showed dependence on the carbon-chain length (CL), double-bond number (DB) and positions (DBP) for FAMEs (supplementary Fig. S4). By using MOMR and best-subset selection approaches, we employed data from 45 representative FAMEs to have established an empirical mathematical model for *t*_*R*_ as a function of CL (*c*), DB (*d*) and DBP (*p*), ${t}_{R}=-6.11{{E}^{-4}c}^{3}+2.56{{E}^{-3}c}^{2}p+ 4.58{E}^{-2}{c}^{2}+8.21{E}^{-3}c{d}^{2}-$$ 0.18{d}^{2}-0.10cp-0.78c+0.22d+0.97p+5.85 $. Five-fold cross-validation indicated the model reliability with good correlation (*R*^2^, 0.9871) and small residual standard error (0.16 min). The model-calculated (*t*_*R*_^*C*^) and experimental retention-time (*t*_*R*_^*E*^) showed nice correlation (*R*^2^ ~0.9899) for these 45 FAMEs (supplementary Fig. 3A). For five test analytes which were not included in the model-building but detected in biological samples, their predicted *t*_*R*_ values were consistent with the measured ones with Δ*t*_*R*_ < 0.38 min (supplementary Table S8, Fig. 3B). Gratifyingly, their measured *t*_*R*_ values from biological samples and analyte standards showed Δ*t*_*R*_ < 0.02 min. Another analyte C22:2n9c was also detected here from some biological samples and assigned to the NIST database though commercial standards were unavailable. Nevertheless, its model-calculated *t*_*R*_ values were consistent (Δ*t*_*R*_ < 0.38 min) with the experimental one as well (supplementary Table S8). Such model-calculation values are expected to be useful for MS quantification in SIM mode and identification of FAs especially when commercial standards are unavailable.

To correct *t*_*R*_ drifts resulting from different temperature gradients, flow rates and acquisition batches, furthermore, we developed a no-additive retention index (NARI) using saturated fatty-acids methyl-esters (SFAMEs) endogenously present in samples instead of spiking alkanes for traditional RI. This is because adding alkanes will inevitably change sample matrices and co-eluted alkanes will inevitably interfere with analyte ionization limiting the use of FID and ion-selection in SIM mode. In this study, minor retention-time variations (Δ*t*_*R*_ < 0.04 min) were observed for all analytes in multiple different matrices (mixed FAME standards, human urine, plasma, H1299C cells, mouse feces and rabbit liver tissue) analyzed in the same batch (supplementary Table S9). In contrast, retention-time variations between different temperature gradients, flow rates and batches were much larger (Δ*t*_*R*_ ~1.6 min) as expected (supplementary Figs. S5A and S6A). Apparently, three different NARI schemes derived from three sets of SFAMEs all showed their powerfulness to correct *t*_*R*_ variations from different temperature gradients, flow rates and batches (supplementary Figs. S5, and S6). Remarkably, NARI from seven FAMEs (C8:0, C16:0, C20:0, C22:0, C24:0, C25:0, C30:0) reduced all above inter-experimental Δ*t*_*R*_ to 0.025 min (supplementary Fig. S6B) which performed better than the alkanes-based RI (Δ*t*_*R*_ < 0.033 min) (supplementary Fig. S6E). Some of these seven analytes might not be detectable, such as C25:0 and C30:0 in urine (supplementary Table S9), or below the limit of quantification in some biological samples (supplementary Table S7). Nevertheless, the *t*_*R*_data for these FAMEs from standard calibration curves within the same batch are still useable with very small intra-batch *t*_*R*_variations (Δ*t*_*R*_ < 0.02 min) resulting from different matrices (Chen *et al.* 2024).

## CONCLUSION

We developed an 8-min GC-FID/MS method for simultaneously quantifying FA composition in biological samples with femtomole-level sensitivity, good accuracy, precision, stability and applicability to biofluids, cells, intestinal contents and tissues. Molecular phenotypes of these biological samples were quantitatively characterized showing unusually high levels of numerous odd-carbon fatty acids in mammalian cells, liver and plasma samples with dietary and gut microbial implications. We also established a no-additive retention index with endogenous analytes to enable comparison of *t*_*R*_ data from different batches. We further established a quantitative structure-retention relationship (QSRR) for FAMEs with variable chain length, double-bond number and positions. To the best of our knowledge, such models have not been reported so far and are useful to predict *t*_*R*_ values for FAMEs with standards unavailable. A number of applications of this rapid quantitative method to large population studies are now undergoing in this lab.

## MATERIALS AND METHODS

### Materials and reagents

All FA standards were purchased from commercial sources with details listed in supplementary Table S10. Potassium carbonate (K_2_CO_3_, 99.5%) was obtained from Aladdin Biochemical Technology Co. Ltd. (Shanghai, China). HPLC-grade methanol (MeOH) and n-hexane were purchased from Sigma-Aldrich (MO, USA) together with acetyl chloride (CH_3_COCl, 99.0%) and butylated hydroxytoluene (BHT, 99.0%). Ultrapure water was prepared by a Milli-Q purification system (Millipore, MA, USA).

### Collection of biological samples

Human blood plasma and urine samples were obtained from Chinese adult volunteers recruited for the Human Phenome Project approved by the Ethics Committee of Fudan University (FE21087) with informed consent from all participants. Human cells, H1299, were acquired from the China Center for Type Culture Collection (CCTCC). Liver tissue samples of New Zealand rabbits were acquired from the Song Lian Experimental Animal Center in Songjiang District (Shanghai, China). Cecum contents of C57BL/6 mice from the Zi Yuan Experimental Animal Science and Technology (Zhejiang, China) were collected according to the National Guidelines for Experimental Animal Welfare (MOST of PR China, 2006). Rabbit liver tissue and cecum contents of C57BL/6 mice were approved by the Experimental Animal Ethics Committee, School of Pharmacy, Fudan University (2018-03-YL-GW-01). All samples were snap-frozen with liquid nitrogen and stored at –80 °C prior to analysis.

### Preparation of stock and working solutions

A stock solution of 45 FA standards was prepared with MeOH at concentrations around 0.17–25.37 mmol/L (supplementary Table S10) with BHT added as an antioxidant. The solution for each of the five test analytes was prepared similarly. The stock solution was then sequentially diluted to obtain standard working solutions for calibration curves and these from 50-, 10- and 2-fold dilution were used as low, medium and high concentration quality control (QC) samples, respectively. A MeOH solution of C17:0-d_33_ (0.10 mmol/L) and C19:0-d_37_ (1.00 mmol/L) was used as internal standards (IS).

### Transesterification of fatty acids in different lipids

We first systematically optimized the MeOH-to-hexane ratio, reaction time (1–3 h) and temperature (60–90 °C) from the previous methods (An *et al.* 2013; Li *et al.* 2015a) to establish an effective FA methylation method. In brief, 15 μL working solutions of FA standards were added with 15 μL IS and 500 μL CH_3_COCl solution (12.5%, *V/V*) in mixed MeOH-hexane (6:1, *V/V*). After 3 h incubation (73 ± 3 °C, 500 r/min) using a TUS-200P incubator (Yi Heng Technology, Shanghai), the mixture was cooled down to room temperature and slowly added with 500 μL K_2_CO_3_ aqueous solution (10%) under ultrasonic condition. An extra 100 μL hexane was then added, vortex-mixed for 2 min and centrifugated (4,500 *g*, 10 min) to obtain the upper layer for GC-FID/MS analysis. For biological samples, the working solution was replaced by 15 μL biofluids (plasma, urine) or about 10 mg intestinal contents or cells or homogenized tissues, respectively. The above procedures were conducted in glass vials to avoid plastic contamination. The efficiency of the above transesterification was monitored with an LC-MS method (Huang *et al.* 2019a; Loo *et al.* 2021) for all 13 lipid subclasses.

### GC-FID/MS analysis

An Agilent 9000 gas-chromatography (GC) system was used for quantification with a flame-ionization detector (FID) and an Agilent 5977B electron-ionization (EI) mass spectrometer (Agilent Technologies, USA). FID and MS signals (at 70 eV) were acquired simultaneously for all analytes. GC separation was accomplished on an Agilent DB-FastFAME capillary column (20 m × 0.18 mm × 0.20 μm) with helium as carrier gas (1.0 mL/min); 1 μL sample was injected and analyzed with the split-ratio of 20:1 and the optimized temperature-gradient listed in supplementary Table S1. The temperature was set to 260, 250, 150 and 230 °C for the injection port, MSD transfer line, quadrupole and ion source, respectively. FID was used for quantifying FAs with more than 24 carbons and MS in selected ion monitoring (SIM) mode for the rest FAs to obtain the best responses.

### Data processing and statistical analyses

Agilent MassHunter Workstation Qualitative and Quantitative Analysis software (v10.1) was used for peak-deconvolution and identification. For MS, one quantifying ion and at least two identifying ions were used for each analyte (supplementary Table S2) to avoid false positives. Fatty acids in biological samples were identified using *t*_*R*_, quantifying and identifying ions together with their abundance ratios obtained from FA standards. The above method was validated for sensitivity, linearity, precision, accuracy and stability (supplementary information notes). All statistical analyses were conducted using R-platform (v 4.1.0) with *p* < 0.05 considered as significant.

### Quantitative structure-retention relationship for FAMES in GC-FID/MS analysis

Experimental *t*_*R*_ values for FAMES from 45 FA standards were employed to construct multivariate-orthogonal multinomial-regression (MOMR) models against their structural characteristics including carbon-chain length (CL), double-bond (DB) number and DB position (DBP). LM function of R-software was used for evaluation with 5-fold cross-validation performed to obtain the model having minimized prediction error. Another six FAs were used as test analytes for assessing prediction accuracy. This enabled coverage of 50 fatty acids with commercial standards.

### No-additive retention index (NARI) for GC-FID/MS analysis of FAMES

Experimental *t*_*R*_ values for the FAMES from selected endogenous FAs were used to establish a retention index (supplementary information notes) to correct *t*_*R*_ variations resulting from different experimental conditions such as temperature-gradient, flow-rate and inter-batch variations. This was done without any exogenous additives. We also built a classical alkanes-based RI in the classical way (Kovats 1958) as a comparison.

## Conflict of interest

Pinghui Liu, Qinsheng Chen, Lianglong Zhang, Chengcheng Ren, Biru Shi, Jingxian Zhang, Shuaiyao Wang, Ziliang Chen, Qi Wang, Hui Xie, Qingxia Huang and Huiru Tang declare that they have no conflict of interest.
